# Effects of focus training on heart rate variability in post-stroke fatigue patients

**DOI:** 10.1186/s12967-022-03239-4

**Published:** 2022-01-31

**Authors:** Yao Wang, Gonglian Xiao, Qing Zeng, Mingjun He, Fei Li, Jiaxin Lin, Xun Luo, Yulong Wang

**Affiliations:** 1 Department of Rehabilitation, Shenzhen Dapeng New District Nan’ao People’s Hospital, Shenzhen, 518121 China; 2 Department of Rehabilitation Medicine, The First Affiliated Hospital, Shenzhen University, ShenZhen Second People’s Hospital, No. 3002 of West Rood, Futian District, Shenzhen, 518060 China; 3 Department of Rehabilitation Medicine, Zhujiang Hospital, Southern Medical University, 253 Industrial Avenue, Guangzhou, 510282 China; 4 Kerry Rehabilitation Medicine Research Institute, Shenzhen, 518048 Guangdong China

**Keywords:** Post-stroke fatigue (PoSF), Self-generate physiological coherence system (SPCS), Heart rate variability

## Abstract

**Objective:**

This study discusses the effects of focus training on heart rate variability (HRV) in post-stroke fatigue (PoSF) patients.

**Methods:**

Self-generate physiological coherence system (SPCS) was used for the focus training of PoSF patients for 12 weeks. Then, fatigue severity scale (FSS), Hamilton depression scale (HAMD), HRV and satisfaction scale (SASC-19) before and after the training were assessed.

**Results:**

Compared with the control group, FSS score, HAMD score, RMSSD, PNN50% were significantly lower in the research group at the end of the intervention (P < 0.05); SDNN, SDANN, LF, HF, LF/HF intervention satisfaction rate increased significantly in the research group at the end of the intervention (P < 0.05).

**Conclusion:**

The use of SPCS software during the focus training of PoSF patients reduced the fatigue and depression, meanwhile improved the HRV of the patients. Therefore, these patients were greatly satisfied with the intervention.

## Introduction

Cerebral stroke has three characteristics of high incidence rate, high disability rate and high mortality. At present, it is the most common disease that causes disability in adults, and the second most common disease that causes death in middle-aged and elderly people [1]. For the treatment of cerebral stroke, except for pharmacological treatment, operation and other methods, present studies have proven that implementing rehabilitations with pertinence for patients has positive meaning in the aspects of relieving extremity function disturbance, lowering disability rate and improving living quality [2]. Post-stroke fatigue (PoSF), one of the common and highly prevalent clinical symptoms after stroke, has been regarded as one of the main complications of stroke patients, with prevalence estimates ranging from 25 to 85%. [3–5]. The occurrence of PoSF not only seriously affects the rehabilitation of post-stroke patients, but also affects the daily life of patients [6]. A recent study has shown that fatigue was still present in one-third of patients until 6 years after stroke onset [7]. Therefore, it is extremely necessary to establish a set of quick and effective rehabilitation schemes for stroke patients.

There is currently insufficient evidence to support any pharmacological or non-pharmacological intervention for the treatment of post-stroke fatigue [8]. Therefore, these is positive meaning in searching for new intervention methods that can help post-stroke patients relieve their negative emotion and feeling of fatigue, improve the rehabilitation training effect, and promote their life quality [9]. The heart rate variability (HRV) is able to predict the degree of fatigue of these patients [10]. In recent years, the application of self-generate physiological coherence system (SPCS) has been given increasing attention and can be considered as a method of focus training. This system is used for mental regulation training and pressure reduction, and this also allows for the collection of the physical and psychological signals of patients, in order to provide patients with specific training and mental regulation intervention. Furthermore, this enables the objects of strengthening the mental regulation ability and improving the physical health. However, there was few report on the application of this system for intervention on heart rate variability (HRV) in PoSF patients [11–13]. These studies were mainly applied to patients with post-stroke depression and found improvements in emotional sleep and HRV [14].

Based on this background, the present study used self-generate physiological coherence system for 60 PoSF patients who received focus training in the last 2 years in our hospital, in order to analyze the effects and influence of SPCS on HRV in patients, and provide guidance in clinical treatment.

## Data and methods

### Study population

After the approval of the Medical Ethics Committee of our hospital, a total of 60 PoSF patients, who received treatment and were cured in our hospital from February 2017 to December 2018, were selected. During the study, none of these patients fell-off or died, and all patients and their family members understood the contents of the study and signed informed consent. All patients complied with the relevant diagnosis standards of the *China Acute Ischemic Cerebral Stroke Diagnosis and Treatment Guideline 2010* [15]. Meanwhile, they also complied with the definition and standard of De Groot MH [16] on PoSF: In the past four weeks, the patients had an obvious feeling of fatigue for continuous two weeks, and these subjective feelings were mainly the feeling of tiredness or exhaustion. These patients did not have significant energy, and needed to increase their time for rest. Their physical activity levels failed to meet the degree of fatigue. In addition, one of the following six items was met: (1) the patients felt that they were not energetic or recovered after having a good rest or sleep; (2) the balance between protective stimulation and cut result was interrupted; (3) the patients thought that they could only overcome the state of inactivity after trying hard enhancement; (4) the patient’s normal life could not be completed or maintained due to the feeling of fatigue; (5) after becoming tired, there was a feeling of discomfort for continuous hours; and (6) the anxiety related to fatigue was extremely obvious. Furthermore, these included patients should also comply with the following standards: (1) within 1–6 months; (2) comply with the above diagnosis criteria related to cerebral stroke; (3) patients with a clear mind are able to cooperate well during the questionnaire survey or voluntarily fill-in the questionnaire or scale; and (4) patients within the age scope of 35–75 years old. Exclusion criteria: (1) patients combined with diseases of the vital organs, such as heart, liver and kidney; (2) patients combined with cardiac arrhythmias; (3) infected patients and patients with serious cognitive disorder or global aphasia, or patients who could not cooperate with researchers; (4) patients who had a HAMD score of ≥ 24 after evaluation, i.e., patients combined with severe depression; (5) patients who had a Epworth Sleepiness Scale score in the range of 10–24; (6) patients used of medication such as Beta blockers, ACE inhibitors, antiarrhythmic drugs, and psychotropic drugs; and (7) patients with active or passive smoking and regular alcohol abuse.

### Groupings

The 60 patients who complied with the criteria were randomly allocated to two (*n* = 30, each group): control group and research group.

According to the literature (Effects of heart rate variability biofeedback therapy on patients with poststroke expression: a case study), the sample size calculated with SDNN index, µ 1 = 44.62, µ 2 = 18.22, σ = 11.27, α = 0.05, β = 0.1. According to the analysis of Pass15 software, the total sample size is 12. The sample size of this study is sufficient.

### Intervention method

All the patients received basic treatment and rehabilitation training for stroke: (1) Basic treatment: cerebral stroke patients with were given grade II preventive treatment, including anti-platelet aggregation according to the actual conditions of the patients, and blood glucose control, blood pressure control, blood lipid control and symptomatic treatment for the other basic diseases; and (2) Rehabilitation training: It included the placement of the position of the affected limbs and good limbs, joint motion, training of muscular strength, balance, and daily life ability. The above training was carried out once a day for 40 min per time from Monday to Saturday. Based on these, SPCS was applied for patients in the research group to carry out the focus training.

SPCS is a new focus training system jointly launched by the Institute of HeartMath and HF Digital Technology Co., Ltd. This mainly involves three independent balance technologies of mind freezing, mind locking, and mind blocking. The system was researched on the basis of the heart and brain interaction theory and pressure reduction theory. It is a new system to adjust and train on HRV, and help patients improve their independent coordination level. Its core mechanism is to take the precise biological feedback sensor as the medium and take the high-tech training software as the carrier, in order to dynamically display the change in HRV of these subjects, and help clinicians more comprehensively understand the balance of the autonomic nerve of these subjects. Moreover, according to the result, this would allow for the design of a reasonable intervention and intensive training plan for patients, in order to help balance the autonomic nerve system of patients, coordinate with and improve the HRV, and change the brain wave activity of these patients to promote the scientific decision-making of the brain. In addition, the focus training carried out by this system will be able to connect the bottom of the frontal lobe from the amygdaloid nucleus of the brain center. The bottom of the frontal lobe is the part for decision-making, emotion and comprehensive intellect. If patients are given a hand at this time to achieve the balance in their autonomic nerve system, and the extraordinary harmoniousness of spirit and physiology, emotions, such as tension, upset, anxiety and depression, can be maximally eliminated. Therefore, the compliance of the rehabilitation treatment can be improved, and the feeling of fatigue can be promoted.

SPCS was provided by Beijing HF Investment Co., Ltd., and the type was HAPPY HEART 2.0. SPCS is a high-tech product based on the HRV autonomous coordination technology and biofeedback technology developed by the Institute of HeartMath. It is composed of precision photoelectric sensor, information processor and high-tech training software. “SPCS” real-time assess HRV (heart rate variability), and use the independent coordinate to adjust the training of HRV. It could interprete the activity password during of heart, brain, and autonomic nervous system. Meanwhile, it is with of “mental training” to balance and improve HRV, that will make users achieve balanced state of autonomic nervous system, and eliminate the negative emotions, such as anxiety, depression, tension, impulse. Because of above all, it will improve the body disease caused by psychological factors, and have mental and physical health [14].

The application procedures were divided into three parts: independent three-step approach learning about balance, HRV evaluation, and HRV strengthening training. The application of the system are presented as follows: installation of the system and entering the system; creating an independent user and logging in; entering the learning center after installing and wearing the equipment; starting the learning of the independent three-step approach. The independent three-step approaches include thought locking (focusing on one thing), respiration resonance (heart rate is equal to twice the breathing rate or some other integer multiple, and the time of adjusting the heart rate coincides with the time of the highest breathing rate), and transference (The process by which the patients put his or her past feelings onto the investigator). Then, they entered the monitoring center to monitor the change in HRV and a state of harmony between mind and body. Afterwards, they entered the training center after confirming the result, and selected games [17]. The types of games included banyan (this training can help patients to quickly enter the state of independent balance, and relieve the negative emotion, such as tension and anxiety), archery (this training can help patients build the ability of independent balance, and facilitates the cultivation of the stability of emotion and the continuity of attention), ingenuity (this training can adjust the independent balance of patients when they are with operation task), quick matching (this training allows patients to complete other tasks at the same time of self-adjustment, and improve their cognitive performance by establishing independent balance), and adventure island (this training can adjust the emotion of a patient under high pressure, and provides a task to keep their focus and establish the ability of independent balance). Lastly, these patients can enter the record center to check the daily training/monitoring results, helping them to be more confident to the post-stroke rehabilitation when they were finding the changes of themselves. The rehabilitation and focus trainings were continuously carried out on a daily basis for 12 weeks, while Sunday was allocated for rest.

### Observation index

#### Improvement of fatigue

The fatigue severity scale (FSS) [18] was used to evaluate and compare the improvement of fatigue in these two patients groups before and after the intervention, respectively. The scale was comprised of nine items. All items were calculated using a score with a range of 1–7, where 1 point means strongly disagree, while 7 points means strongly agree, and the total score was 63. When the score was ≥ 27, this indicated that there was fatigue. The higher the score was, the more intensive the feeling of fatigue of these patients was.

#### Depression

The Hamilton Depression Scale (HAMD) [19] was used to evaluate the depression of patients before and after treatment, respectively. This scale was comprised of 17 questions, and the total score was 54. When the total score was < 7, there was no depression. When the total score was 7–17, there may be depression. When the total score was 17–24, there must be depression. When the total score was > 24, this means that there is serous depression.

#### Heart rate variability

The Holter-star 24-h Comprehensive Information dynamic electrocardiogram system provided by Langang Electronic Instruments Co., Ltd. was used to monitor the patients' HRV before and after treatment. These indexes included time domain indexes and frequency domain indexes. The domain indexes included the standard deviation of 24-h normal R-R interval (SDNN), the mean standard deviation of R-R interval every five minutes in 24 h (SDANN), the mean the square root of difference between adjacent R-R intervals at 24 h (RMSSD), and the 24-h adjacent R-R interval difference of > 50 ms percentage (pNN50%). The frequency domain indexes include 0.04–0.15 Hz low frequency (LF), 0.15–0.4 Hz high frequency (HF), and LF/HF.

#### Satisfaction

The treatment satisfaction scale (SASC-19) of cerebral stroke patients was established by referring to the study conducted by HAN B, et al. [17]. The questionnaire had eight items, and each item was calculated using a 4-level point system: 0 point means strongly disagree; 1 point means basically agree; 2 points means agree; and 3 points means strongly agree. The total score was 24. Furthermore, a score of ≥ 20 indicates strongly satisfactory; a score within 14–19 indicates satisfactory; and a score of < 14 indicates dissatisfactory. Total satisfaction = very satisfactory + satisfactory.

### Statistical method

SPSS 20.0 statistical software was used to the process data. The data before and after intervention were compared by paired t-test. X refer to mean value, S refer to standard deviation. Mean ± SD was used to indicate the measurement data. And it was inspected by *t*-test. Percentage was used to indicate the enumeration data, it was inspected by *χ*^*2*^ and a normal distribution test was performed. *P* < 0.05 was considered statistically significant.

## Results

### Patients

A total of 60 patients were enrolled onto the study, including 30 in the control group and 30 in the research group. All patients completed the experiment as planned.

**Fig. 1:**
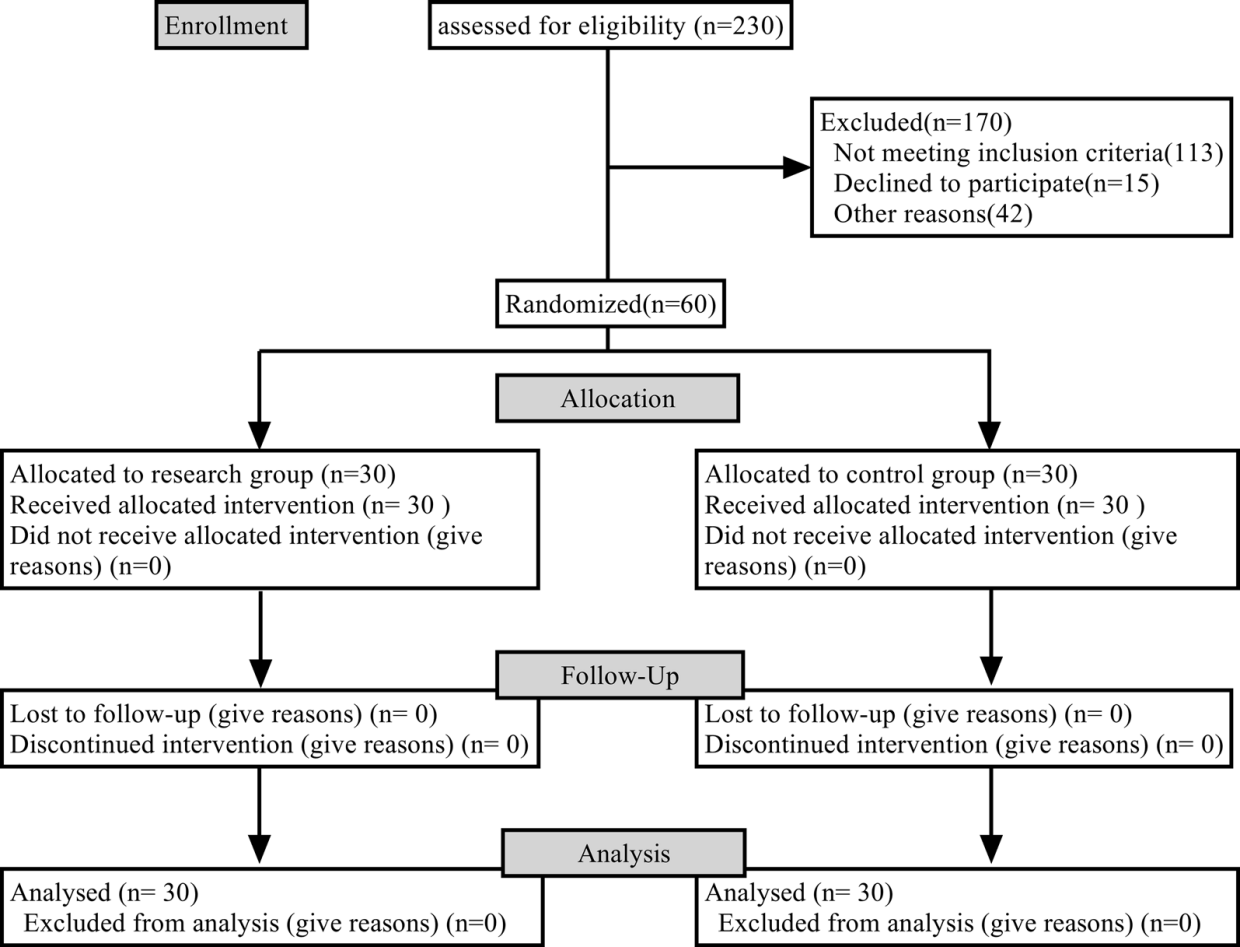
Flow chart of study process

There were 20 male and 10 female patients in the control group. The age of these patients ranged within 40–75 years old, with an average age of 54.15 ± 9.57 years old, and the course of disease was 15–90 days, with an average of 44.45 ± 15.02 days. There were 19 male and 11 female patients in the research group. The age of these patients ranged within 42–75 years old, with an average age of 54.02 ± 9.48 years old, and the course of disease was 15–90 days, with an average 45.02 ± 15.11 days. Analysis of the general materials between two groups, such as gender, age and course of disease, the difference was not statistically significant (*P* > 0.05) and the two groups were comparable (Fig. 1).

### FSS and HAMD scores

After intervention, the fatigue severity scale scores of patients in both two groups were lower than those before intervention. Furthermore, the reduction in the research group was higher than that in the control group, and the difference was statistically significant (*P* < 0.05, Table 1).

**Table 1:** Comparison of FSS and HAMD scores in the two groups ($\overline{x}$ ± *s*, score)

Group	FSS Score		HAMD Score	
	Before intervention	After intervention	Before intervention	After intervention
Research group (n = 30)	43.02 ± 9.51	23.12 ± 7.45^a^	20.14 ± 3.21	10.21 ± 3.24^a^
Control group (n = 30)	42.96 ± 9.02	30.17 ± 6.59^a^	20.09 ± 3.17	15.14 ± 5.11^a^
*t*	0.025	3.882	0.061	4.463
*p*	0.980	< 0.001	0.952	< 0.001

To compared with the same group before the intervention

FSS, the fatigue severity scale; HAMD, the hamilton depression scale

^a^*P* < 0.05

### Heart rate variability

#### Time domain indexes

After intervention, the SDNN and SDANN values of patients in these two groups increased than before intervention, while RMSSD and pNN50 decreased than before intervention. In addition, the increase/reduction amplitude of indexes in the research group were higher than those in the control group, and the difference was statistically significant (*P* < 0.05, Table 2).

**Table 2:** Comparison of HRV time domain indexes in the two groups ($\overline{x}$ ± *s*)

Group	Research group (n = 30)	Control group (n = 30)	*t*	*p*
SDNN (ms)				
Before intervention	43.12 ± 7.01	42.59 ± 6.89	0.295	0.769
After intervention	109.45 ± 7.45^a^	80.12 ± 5.69^a^	17.137	< 0.001
SDANN (ms)				
Before intervention	36.98 ± 5.12	37.01 ± 6.02	0.021	0.984
After intervention	95.52 ± 9.56^a^	65.11 ± 8.65^a^	12.919	< 0.001
RMSSD (ms)				
Before intervention	14.29 ± 4.67	14.32 ± 4.72	0.025	0.98
After intervention	3.32 ± 1.74^a^	7.02 ± 2.11^a^	7.411	< 0.001
pNN50				
Before intervention	6.41 ± 1.79	6.33 ± 1.82	0.172	0.864
After intervention	1.29 ± 0.79^a^	3.46 ± 1.02^a^	9.213	< 0.001

To compared with the same group before the intervention

SDNN, the standard deviation of 24-h normal R-R interval; SDANN, the mean standard deviation of R-R interval every five minutes in 24 h; RMSSD, the mean the square root of difference between adjacent R-R intervals at 24 h; pNN50, the 24-h adjacent R-R interval difference of > 50 ms percentage

^a^*P* < 0.05

#### Frequency domain indexes

After intervention, the LF, HF and LF/HF of patients in these two groups increased than before intervention, The increase amplitude in the research group was higher than that in the control group, and the difference was statistically significant (*P* < 0.05, Table 3).

**Table 3:** Comparison of HRV frequency domain indexes in the two groups ($\overline{x}$ ± *s*)

Group	Research group (n = 30)	Control group (n = 30)	*t*	*p*
LF (ms^2^/Hz)				
Before intervention	467.85 ± 101.69	468.95 ± 103.24	0.042	0.967
After intervention	988.46 ± 245.46^a^	600.69 ± 132.48^a^	7.615	< 0.001
HF (ms^2^/Hz)				
Before intervention	440.46 ± 214.55	451.12 ± 222.01	0.189	0.851
After intervention	677.79 ± 201.24^a^	506.45 ± 220.14^a^	3.147	0.003
LF/HF				
Before intervention	1.10 ± 0.80	1.09 ± 0.78	0.049	0.961
After intervention	1.94 ± 0.71^a^	1.17 ± 0.82^a^	3.686	0.001

To compared with before the intervention

LF: 0.04–0.15 Hz low frequency; HF: 0.15–0.4 Hz high frequency

^a^*P* < 0.05

### Satisfaction

The total satisfaction for the intervention was higher in the research group than in the control group, and the difference was statistically significant (*P* < 0.05, Table 4).

**Table 4:** Comparison of intervention satisfaction between the two groups n (%)

Items	Research group (n = 30)	Control group (n = 30)	*χ*^2^	*p*
Great satisfaction	20 (66.67)	10 (33.33)	–	–
Satisfaction	9 (30.00)	12 (40.00)	–	–
Dissatisfaction	1 (3.33)	8 (26.67)	–	–
Total satisfaction	29 (96.67)	22 (73.33)	4.706	0.03

## Discussion

In this study, we found that the use of SPCS software during the focus training of PoSF patients reduced the fatigue and depression, meanwhile improved the HRV of the patients. Therefore, these patients were greatly satisfied with the intervention.

The main cause of cerebral stroke is blood supply disorder of the brain, which leads to nerve cell damage, and the decline and even loss of the corresponding functions. This is a kind of disease that can induce language, action and emotion disorders. After its occurrence, patients often feel fatigue, which not only reduces their life quality, but also brings a certain negative emotion to patients, such as depression and anxiety [20].

Post stroke fatigue is complex and multidimensional, including emotional, cognitive, physical and social aspects. The accurate diagnosis of post-stroke fatigue and the auxiliary diagnosis suitable for instruments are still not ideal [21]. In addition, stroke patients may not be immediately aware of their fatigue, which are challenges we need to face.

The renin angiotensin system (RAS), a key regulator of blood pressure and fluid balance [22], is an important humoral regulatory system composed of a series of peptide hormones and corresponding enzymes. Inappropriate activation of intrarenal RAS is an important mechanism involved in the pathogenesis of hypertension and nephropathy [23]. It is noteworthy that hypertension is the most important independent risk factor for stroke, so the renin-angiotensin system in patients with post-stroke fatigue presents a pathological state.

Studies have suggested that the process of generating negative emotion, such as anxiety and depression, is correlated to the neuroregulation mechanism of the sympathetic nerve and autonomic nerve. Furthermore, there are also studies indicating that the autonomic adjustment of the HRV of patients is abnormal [24, 25]. HRV reflects the autonomic nerve function of the heart, and at post-stroke, HRV decreases. The study conducted by Bodapati et al. [26] indicated that HRV can predict the risk of stroke. In addition, HRV is correlated to neurological function deterioration and recurrence at the early stage of acute ischemic post-stroke [27]. The study conducted by Li et al. [28] revealed that as autonomic nerve functions decrease in depressed patients, the risk of suffering from cardiovascular disease increases. Therefore, performing a reasonable intervention that could help patients lower their sympathetic nerve activity has important meaning for improving vagus nerve tone and adjusting the balance between the sympathetic nerve and vagus nerve. In addition, the study conducted by Li et al. [29] revealed that after the increase in balance of body sympathetic nerve and vagus nerve, and the increase in HRV, the prognosis of cardiovascular and cerebrovascular disease patients obviously improves.

SDNN mainly reflects the tone of the vagus nerve and sympathetic nerve, and is used to evaluate the recovery and damage of the autonomic nerve system. Furthermore, RMSSD and pNN50 are the indexes that reflect the quick change of HRV, such as the indexes of sympathetic nerve tone sensitivity, while SDANN reflects the slow change in HRV, such as the size of the sympathetic nerve tone [30, 31]. The result of the present study indicates that after the focus training of SPCS, the depression of patients in the research group was obviously relieved, when compared to patients in the control group. Furthermore, their HRV indexes also obviously decreased, when compared to patients in the control group. HRV can be used as the biomarker in post-stroke complication and the functional recovery of post-stroke [32, 33]. The patients’ fatigue symptoms obviously reduced as well, this improving the adjustment of the relevant neurotransmitter of these patients may be the cutting point for reducing the feeling of fatigue of PoSF patients [34]. This indicates that the use of SPCS on PoSF patients facilitated the relief in negative emotion of these patients, the HRV of these patients had a better effect, and the effect on improving the fatigue symptoms of these patients was better. In addition, the present study also revealed the satisfaction of patients in these two groups, in terms of the different intervention methods. These results indicate that the total satisfaction was higher in the research group than in the control group, indicating that the application of SPCS made it easier to gain the support and faith of these patients, and the satisfaction of these patients was higher.

In conclusion, the application of SPCS in focus training for PoSF patients can facilitate the relief in the degree of fatigue of patients, improve the effect on the HRV of patients, enable the autonomic nerve function of these patients to be better adjusted, and better improves the depressive state. Furthermore, these patients had high satisfaction with this intervention and the receptiveness is good.

The limitations of the study was that we used not a large number of samples, so the results may not be that convincing. In the further study, geographic differences in population and physical condition are also important factors to be considered so as to explore which factors could affect the results. Moreover, in recent years, nanomedicine, such as calcium carbonate (CaCO_3_) nanoparticles (NP_S_), has attracted much attention in the application of cancer because it can target drug/gene delivery and has the advantages of low cost, safety, biocompatibility and pH-sensitivity [35]. In future research, it may be possible to combine focused training with new nanomedicine to observe the effect of heart rate variability in patients with post-stroke fatigue.

## Data Availability

The datasets used and/or analysed during the current study available from the corresponding author on reasonable request.

## Abbreviations

HRV: Heart rate variability
PoSF: Post-stroke fatigue
SPCS: Self-generate physiological coherence system
FSS: Fatigue severity scale
HAMD: Hamilton depression scale
SASC-19: Satisfaction scale
SDNN: Standard deviation of 24-h normal R-R interval
SDANN: Standard deviation of R-R interval every five minutes in 24 h
LF: Low frequency
PNN50%: R-R interval difference of > 50 ms percentage
RMSSD: Square root of difference between adjacent R-R intervals at 24 h
